# Severe Edematous Facial Myositis Following Dual Influenza and COVID-19 Vaccination: A Case Report

**DOI:** 10.7759/cureus.76540

**Published:** 2024-12-28

**Authors:** Latifa Aljaber, Peter Schutz, Daniel Ennis, Kristin Jack, Kun Huang

**Affiliations:** 1 Internal Medicine, University of British Columbia, Faculty of Medicine, Vancouver, CAN; 2 Pathology, University of British Columbia, Faculty of Medicine, Vancouver, CAN; 3 Rheumatology, University of British Columbia, Faculty of Medicine, Vancouver, CAN; 4 Neurology, University of British Columbia, Faculty of Medicine, Vancouver, CAN

**Keywords:** covid-19 vaccine side-effects, facial angioedema, immunosuppressant therapy, inflammatory myositis, mrna covid-19 vaccination

## Abstract

Idiopathic inflammatory myopathies (IIM), or myositis, are a heterogeneous group of autoimmune disorders that can affect multiple organs, including the muscles, skin, joints, lungs, heart, and gastrointestinal tract. While new-onset myositis has been reported following SARS-CoV-2 infection, cases associated with COVID-19 vaccination remain rare. We describe a unique case of severe progressive edematous facial myositis resembling angioedema in a 22-year-old man, with onset one to two weeks after receiving dual SARS-CoV-2 and influenza vaccinations. This ultimately led to a diagnosis of systemic inflammatory myositis with extensive involvement of proximal muscles in the arms and legs. We outline the clinical course, diagnostic investigations, and treatments, and discuss the potential molecular mechanisms and existing literature on inflammatory myositis induced by SARS-CoV-2 infection or mRNA-based vaccination.

## Introduction

Idiopathic inflammatory myopathies (IIM) are a group of disorders characterized by muscle inflammation leading to weakness, often accompanied by distinctive skin manifestations. These conditions can also affect organs such as the lungs, joints, gastrointestinal system, and heart. Subtypes include dermatomyositis, antisynthetase syndrome, immune-mediated necrotizing myopathy, and overlap myositis, which are classified based on histopathology, specific autoantibodies, and patterns of organ involvement [1]. Although the exact etiology is unknown, environmental factors, viruses, drugs, and vaccines may act as triggers in genetically predisposed individuals [1].

Both SARS-CoV-2 infection and mRNA-based vaccination have been associated with the onset of dermatomyositis (DM) [2-5]. While mild facial or periorbital edema is a recognized feature of DM, a vaccine-induced presentation resembling severe angioedema with facial muscle involvement, culminating in the diagnosis of widespread inflammatory myositis, has not been previously reported.

## Case presentation

We report a case of a 22-year-old previously healthy East Asian male who presented with painless, subacute, progressive periorbital and facial swelling. Symptoms began one to two weeks after dual vaccination with SARS-CoV-2 (Moderna SPIKEVAX Bivalent Original/Omicron) and influenza (Fluzone Quadrivalent). He had no history of regular medication use, new medications, supplements, or recreational drugs. He had previously received annual influenza vaccinations and two doses of Pfizer SARS-CoV-2 vaccines without adverse effects.

**Figure 1:**
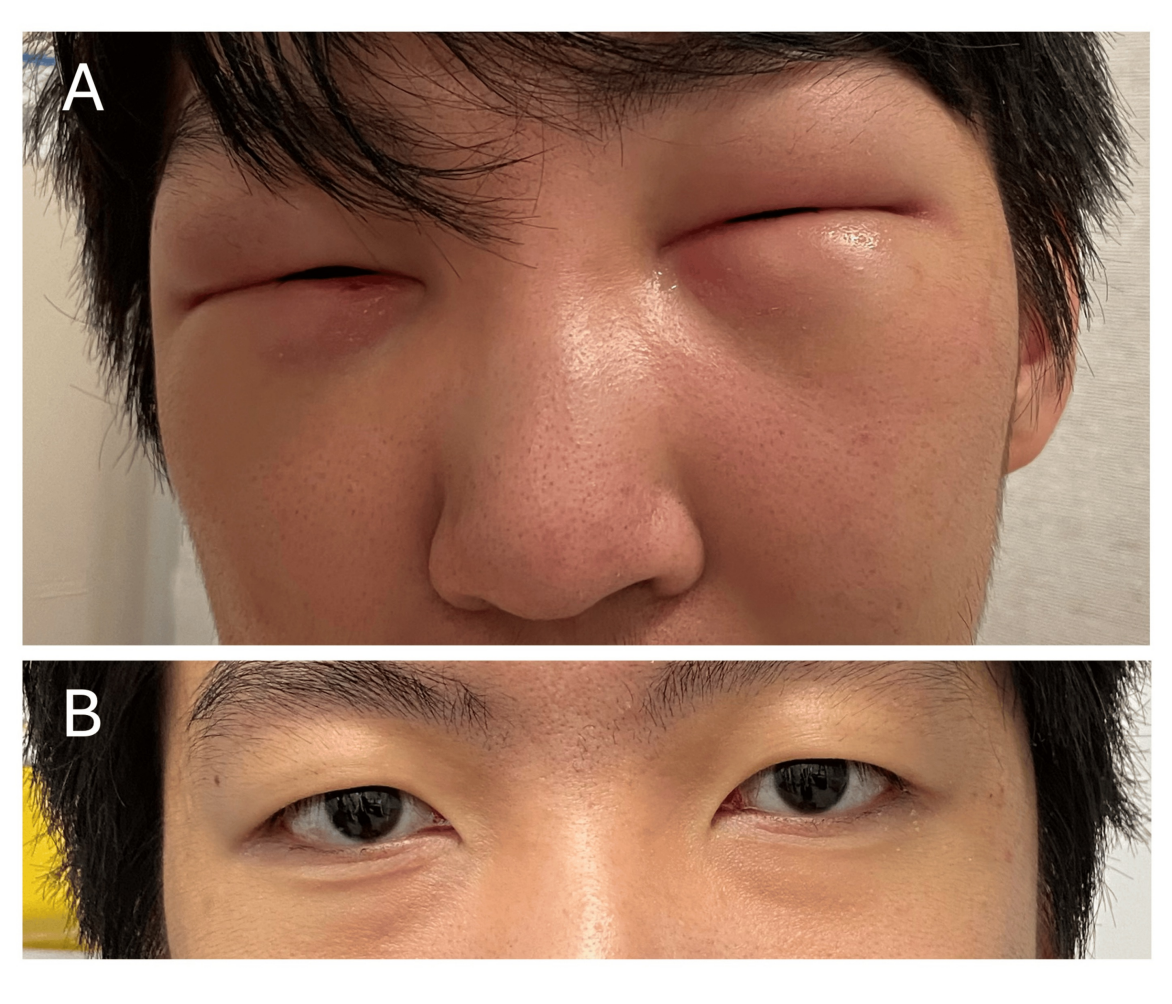
Photos of periorbital and facial edema at presentation and resolution after treatment. (A) Severe periorbital and facial swelling on presentation, causing inability to open the eyes. (B) Complete resolution of swelling one year post-treatment.

Due to worsening facial edema over six weeks, he received an empiric course of prednisone (50 mg daily, tapered over 10 days) for presumed angioedema, resulting in transient improvement. However, symptoms recurred and worsened a few days after prednisone discontinuation, prompting hospital admission (Figure 1A). Review of systems and physical examination were unremarkable except for subtle right triceps weakness. Notably, there were no infectious prodromes, fever, rash, joint pain, or airway/tongue swelling.

On admission, laboratory tests (Table 1) revealed leukopenia, thrombocytopenia, elevated transaminases, ferritin, creatine kinase (CK), and soluble interleukin-2 receptor (sIL-2R). Autoimmune serologies, including antinuclear antibody (ANA), extractable nuclear antigen (ENA), double-stranded DNA (dsDNA), antineutrophil cytoplasmic antibody (ANCA), immunoglobulin G4 (IgG4), and serum protein electrophoresis (SPEP), were negative. Myositis-specific and associated antibodies, including anti-Jo-1, Mi2, MDA5, TIF1γ, and others, were negative. Extensive infectious workup, including for SARS-CoV-2, cytomegalovirus (CMV), Epstein-Barr virus (EBV), HIV, and tuberculosis, was also negative.

**Table 1:** Laboratory data on admission SPEP: serum protein electrophoresis, CK: creatine kinase, MCV: mean corpuscular volume, ANA: antinuclear antibody, ENA: extractable nuclear antigen, dsDNA: double-stranded DNA, ANCA: antineutrophil cytoplasmic antibody, Ig: immunoglobulin

Laboratory parameters	Values	Laboratory parameters	Values
WBC x 10^9^/L	2.6	C-reactive protein g/L	2.2
Hemoglobin, g/L	130	Calcium mmol/L	2.10
MCV, fL	80	Trop I ng/L	6
Platelet x 10^9^/L	88	Triglyceride mmol/L	3.30
Creatinine, umol/L	70	ANA	0.2
Aspartate Aminotransferase (AST), U/L	264	ENA	negative
Alanine aminotransferase (ALT), U/L	319	dsDNA	negative
Alkaline phosphatase (ALP), U/L	73	C3 g/L	1.04
Gamma-glutamyl transferase (GGT), U/L	27	C4 g/L	0.52
SPEP	Normal pattern	ANCA	negative
Urinalysis	Bland, no proteinuria	IgG g/L	8.3
Ferritin ug/L	8430	IgA g/L	1.83
Soluble IL-2 receptor U/mL	8126	IgM g/L	1.41
CK IU/L	6414	IgG4 g/L	<0.006
Lactate dehydrogenase (LDH) u/L	815		

**Figure 2:**
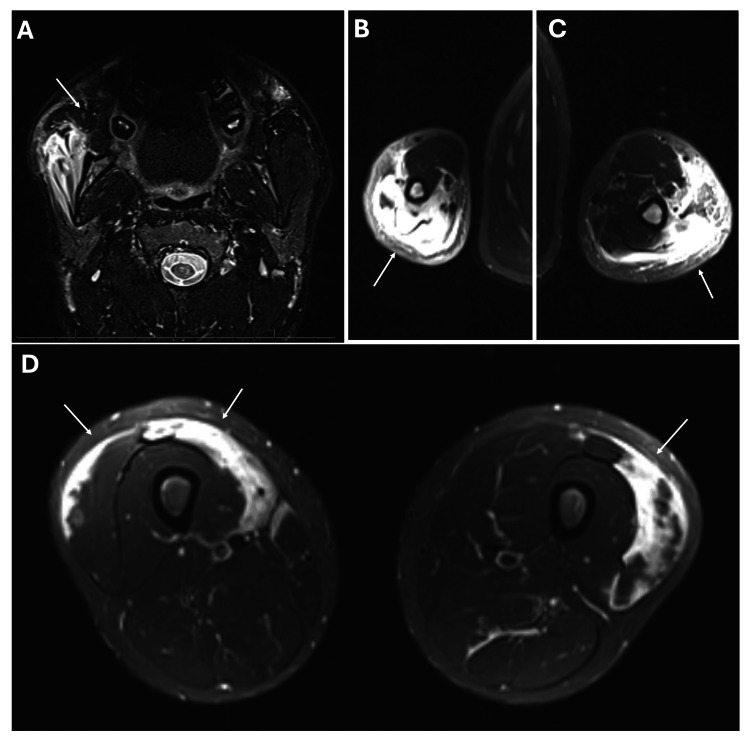
MRI of the facial, humeral and femoral muscles. (A) MRI of the facial muscles revealed thickening, edema, and enhancement of the right masseter muscle (arrow). (B and C) MRI of the upper extremities showed extensive intramuscular, fascial and subcutaneous edema in the triceps (arrows). (D) MRI of the thighs revealed diffuse muscle edema was evident in bilateral vastus lateralis and rectus femoris (arrows).

MRI revealed thickening, edema, and enhancement of the right masseter and bilateral sternocleidomastoid muscles, along with inflammatory changes (Figure 2A). Although weakness was minimal (right triceps only), MRI showed extensive edema in the triceps, forearm muscles, and bilateral quadriceps, with intramuscular hemorrhage (Figure 2B-2E).

**Figure 3:**
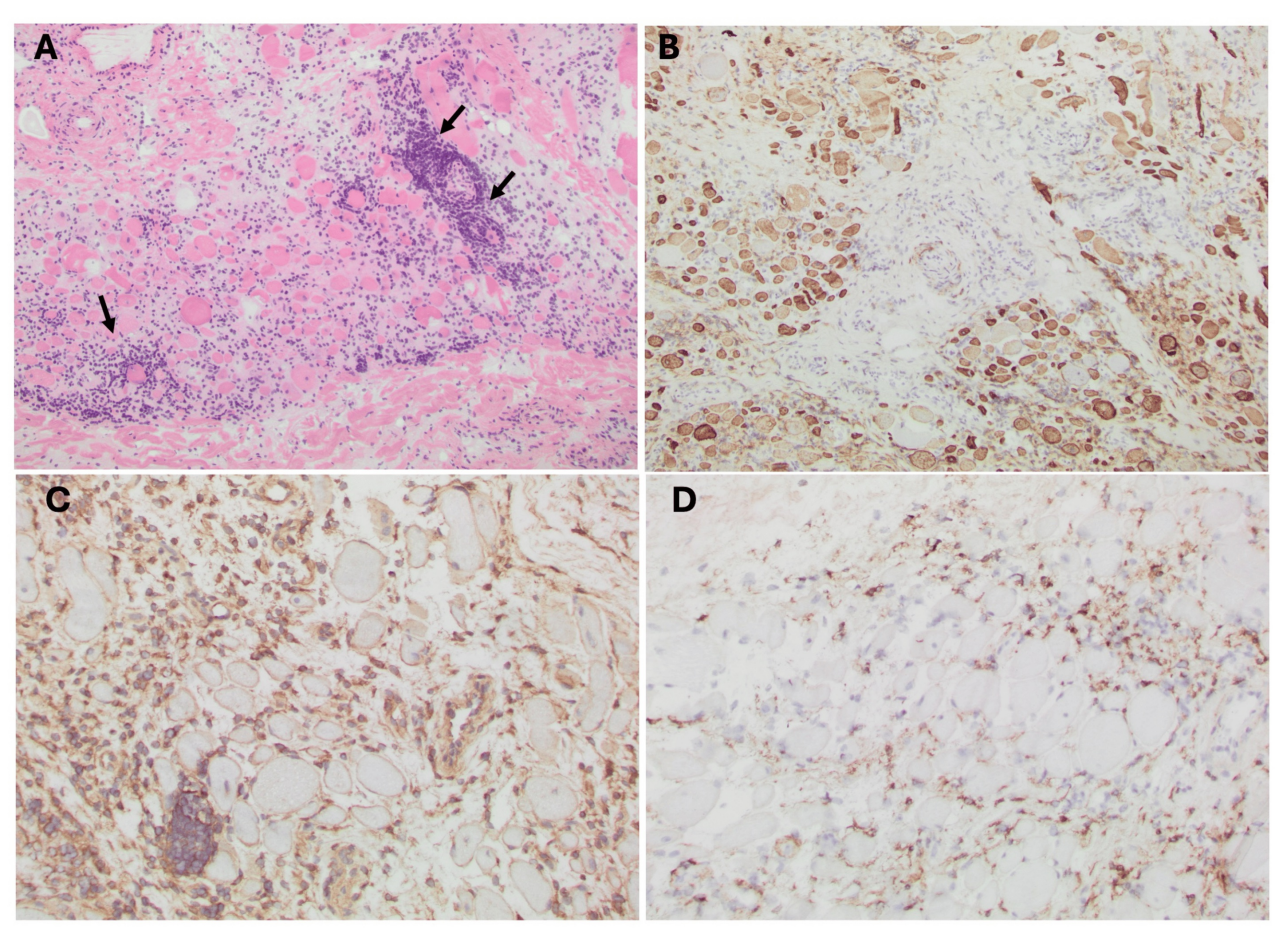
Right triceps muscle biopsy findings (A, B) Extensive endomysial and perivascular lymphocytic infiltrates (arrows) (H&E 10X), predominantly T-cells with a large proportion of CD8+ cells, numerous macrophages, fiber size variability with multiple small regenerating fibers. (C) Diffuse MHC class I upregulation (20X). (D) Negative MxA staining (10X). MHC: major histocompatibility complex, MxA: myxovirus resistance protein A

Muscle biopsy of the right triceps revealed inflammatory myopathy with extensive endomysial and perivascular lymphocytic infiltrates (predominantly CD8+ T-cells and macrophages), fiber size variability, regenerating fibers, and diffuse major histocompatibility complex (MHC) class I upregulation (Figure 3A-3C). No perifascicular accentuation or myxovirus resistance protein A (MxA) positivity was observed (Figure 3D), ruling out dermatomyositis.

Treatment included prednisone (50 mg daily) and methotrexate (25 mg subcutaneously weekly), leading to resolution of edema and normalization of CK. However, at month five, tapering prednisone to 12.5 mg daily resulted in recurrence of facial swelling and CK elevation (741 IU/L). Mycophenolate (1.5 g twice daily) was added, achieving steroid-free remission with normalized CK, strength, and resolution of MRI findings (Figure 1B).

This case represents inflammatory myositis with severe periorbital edema mimicking angioedema, high CK, and intramuscular hemorrhage, but preserved strength. The absence of MxA staining and myositis-specific autoantibodies ruled out known serological phenotypes, such as dermatomyositis or antisynthetase syndrome.

Given the temporal association between vaccination and his symptom onset, extensive negative infectious and autoimmune workup, and global case reports of new onset of DM following SARS-CoV-2 vaccination, we hypothesize the SARS-CoV-2 vaccine as the likely trigger for this severe edematous inflammatory myositis.

## Discussion

Cases of newly developed myositis following COVID-19 vaccination remain rare. Reported subtypes include DM, antisynthetase syndrome, and immune-mediated necrotizing myopathy (IMNM) [6]. Myositis-specific autoantibodies such as anti-MDA5, TIF1γ, SRP, NXP2, Mi-2, and Pm/Scl-75 have been identified following vaccination [6]. Among these, anti-MDA5 has garnered particular interest due to its clinical and pathological parallels with both anti-MDA5+ DM and severe COVID-19 infection [7]. For instance, in Yorkshire, UK, the rate of anti-MDA5 positivity surged following the 2021 peak of COVID-19 cases and coincided with the vaccination rollout [7].

MDA5 is an RNA sensor and a key pattern recognition receptor for SARS-CoV-2 or mRNA [8]. Its encoding gene, IFIH1, is highly upregulated in both COVID-19 infection and autoimmune interstitial lung disease (ILD) [7]. MDA5 activation, whether triggered by infection, vaccination, or both, induces type I interferon (IFN1) production and antigen-specific CD8+ T cell responses [8]. This is particularly relevant in DM, where IFN1 activation is a hallmark of disease pathophysiology.

In contrast, our patient tested negative for myositis-specific autoantibodies and exhibited no histopathological features of IFN1 pathway activation, as evidenced by negative sarcoplasmic MxA staining. Instead, the markedly elevated ferritin, soluble IL-2 receptor (sIL-2r), and cytopenias suggest activation of the IFN2 pathway, characterized by increased interferon-gamma (IFN-γ). These cytokines play a crucial role in cellular immune responses to SARS-CoV-2 vaccination [9].

Treatment for COVID-19 vaccine-induced inflammatory myositis often requires high-dose corticosteroids combined with immunosuppressants, as was the case with our patient. Unlike self-limiting post-vaccine adverse effects, inflammatory myositis typically necessitates sustained immunosuppressive therapy. Treatments reported in the literature include corticosteroids, mycophenolate, azathioprine, cyclophosphamide, tofacitinib, and rituximab [3].

Two questions remain to be elucidated by future observational studies. We do not have any evidence to address whether long-term immunosuppressants are necessary to maintain remission for COVID-19 vaccination-induced inflammatory myositis. It is also of great importance for clinicians to understand the risk of myositis recurrence if such patients are exposed to another dose of mRNA-based COVID-19 vaccination.

## Conclusions

In summary, we report a rare case of severe edematous facial myositis presenting with angioedema and asymptomatic extremity involvement, associated with COVID-19 vaccination. Timely diagnosis and treatment are essential, as life-threatening manifestations of myositis have been reported in similar contexts. While association does not equate to causation, this case highlights the importance of understanding the complex immune interactions between viruses, vaccines, and the host immune system.
